# The Symbiosome: Legume and Rhizobia Co-evolution toward a Nitrogen-Fixing Organelle?

**DOI:** 10.3389/fpls.2017.02229

**Published:** 2018-01-22

**Authors:** Teodoro Coba de la Peña, Elena Fedorova, José J. Pueyo, M. Mercedes Lucas

**Affiliations:** ^1^Instituto de Ciencias Agrarias ICA-CSIC, Madrid, Spain; ^2^Centro de Estudios Avanzados en Zonas Áridas (CEAZA), La Serena, Chile; ^3^K. A. Timiryazev Institute of Plant Physiology, Russian Academy of Science, Moscow, Russia

**Keywords:** endosymbiosis, legumes, rhizobia, nodule, symbiosome, lupin, nitrogen fixation, organelle evolution

## Abstract

In legume nodules, symbiosomes containing endosymbiotic rhizobial bacteria act as temporary plant organelles that are responsible for nitrogen fixation, these bacteria develop mutual metabolic dependence with the host legume. In most legumes, the rhizobia infect post-mitotic cells that have lost their ability to divide, although in some nodules cells do maintain their mitotic capacity after infection. Here, we review what is currently known about legume symbiosomes from an evolutionary and developmental perspective, and in the context of the different interactions between diazotroph bacteria and eukaryotes. As a result, it can be concluded that the symbiosome possesses organelle-like characteristics due to its metabolic behavior, the composite origin and differentiation of its membrane, the retargeting of host cell proteins, the control of microsymbiont proliferation and differentiation by the host legume, and the cytoskeletal dynamics and symbiosome segregation during the division of rhizobia-infected cells. Different degrees of symbiosome evolution can be defined, specifically in relation to rhizobial infection and to the different types of nodule. Thus, our current understanding of the symbiosome suggests that it might be considered a nitrogen-fixing link in organelle evolution and that the distinct types of legume symbiosomes could represent different evolutionary stages toward the generation of a nitrogen-fixing organelle.

## Introduction

Symbiosis between different organisms has played a key role in evolution and in fact, the term “symbiogenesis” is an evolutionary concept that refers to “the appearance of new physiologies, tissues, organs, and even new species as a direct consequence of symbiosis” (Chapman and Margulis, 1998; Margulis and Chapman, 1998; O'Malley, 2015). Endosymbiosis is a reciprocal advantageous association in which one organism lives inside another and it has a pivotal importance in symbiogenesis. Endosymbiotic theories to explain the origin of eukaryote cells and their organelles have been proposed and discussed for more than a century (Zimorski et al., 2014; Martin et al., 2015; O'Malley, 2015). Mitochondria and chloroplasts of eukaryotic cells, key organelles for respiration and photosynthesis, are thought to result from the evolution of an ancient endosymbiosis in which ancient bacterial-like organisms were engulfed into an ancient prokaryotic or eukaryotic-like cell (Dyall et al., 2004; Kutschera and Niklas, 2005; Zimorski et al., 2014; Archibald, 2015).

The endosymbiosis that leads to organelle formation follows distinct key processes and stages: recognition between symbionts, engulfment, the failure of defense systems to eliminate the endosymbiont by defense reaction, physiological integration and finally, genetic integration (Margulis and Chapman, 1998). It is commonly accepted that during the transition from an endosymbiont to an organelle, cyclical endosymbiosis becomes permanent or obligate endosymbiosis by the transfer of endosymbiont genes to the nucleus of the host cell, establishment of a protein targeting system to reimport the products of these genes, division of the endosymbiont inside the macrosymbiont and the vertical transmission to the macrosymbiont's offspring (Cavalier-Smith and Lee, 1985; Chapman and Margulis, 1998; McFadden, 1999; Parniske, 2000; Douglas and Raven, 2003; Dyall et al., 2004). Therefore, is it obvious what differentiates an endosymbiont from an organelle? It has been suggested that “the boundaries between these terms can blur” and that it might be necessary to employ other criteria to distinguish an endosymbiont from an organelle (Keeling and Archibald, 2008). Thus, studies focusing on more modern endosymbioses might reveal how organelles came to be and why they look the way they do (Keeling et al., 2015; McCutcheon, 2016).

The oxygen respiration and photosynthetic capacity of ancestral mitochondria and chloroplasts, respectively, was the key driving force for endosymbiosis and co-evolution toward organelle formation. As nitrogen is an important component of biomolecules and frequently a limiting nutrient, nitrogen fixation is a fundamental process in ecosystems (Tyrrell, 1999). The capacity to fix atmospheric nitrogen (diazotrophy) is exclusive to prokaryotic organisms that contain the nitrogenase enzyme complex. Diazotrophs include some archaea and within the eubacteria, they include proteobacteria, cyanobacteria, and actinobacteria. Eukaryotic organisms are unable to fix nitrogen and thus, different types of symbiotic relationships have been established between eukaryotes and diazotrophic bacteria to fulfill this function, ranging from loose interactions to highly regulated intracellular symbioses (Kneip et al., 2007). In these interactions, eukaryotic organisms supply nutrients and energy to the diazotrophs in exchange for fixed nitrogen.

In plants, there are two types of associations with soil diazotroph eubacteria that are relevant to the symbiotic fixation of atmospheric nitrogen in a new organ developed in plant, the nodule. The filamentous Gram-positive bacteria *Frankia* are nitrogen-fixing endosymbionts of plants that are collectively called actinorhizal plants. By contrast, Gram-negative bacteria known as rhizobia, fix nitrogen in root nodules of legumes and of the non-legume *Parasponia*. Nitrogen-fixing symbiosis in legume root nodules is the best studied to date and it is significantly important for the nitrogen input in both agricultural and natural ecosystems. The legume root nodule was considered as “the best example of symbiospecific morphogenesis” (Chapman and Margulis, 1998). Specific recognition between symbionts takes place through the exchange of signaling molecules. For example, legume roots secrete flavonoids and other compounds to the rhizosphere, generally inducing the synthesis and secretion of rhizobial lipo-chito-oligosaccharides (LCOs, Nod factors). These molecules act as mitogens inducing cell division in the root cortex, and the formation of the root nodule through the progressive differentiation of specialized cells and tissues (Pueppke, 1996; Geurts et al., 2005; Cooper, 2007). Concomitant with nodule primordium development, bacteria enter the root cortex and infect cells of the nodule primordium (Brewin, 1991; Jones et al., 2007).

Two main types of symbiotic nodules have been described as a function of the type of growth: indeterminate and determinate. The typical indeterminate nodule is originated by proliferation of inner root cortical cells; it has a persistent apical meristem and adopting a cylindrical shape. The typical determinate nodule originates by proliferation of outer cortical cells and it has a lateral meristem that remains active for some days. After the arrest of meristematic activity, the nodule grows by cell expansion and it adopts a spherical shape (Patriarca et al., 2004).

**Figure 1:**
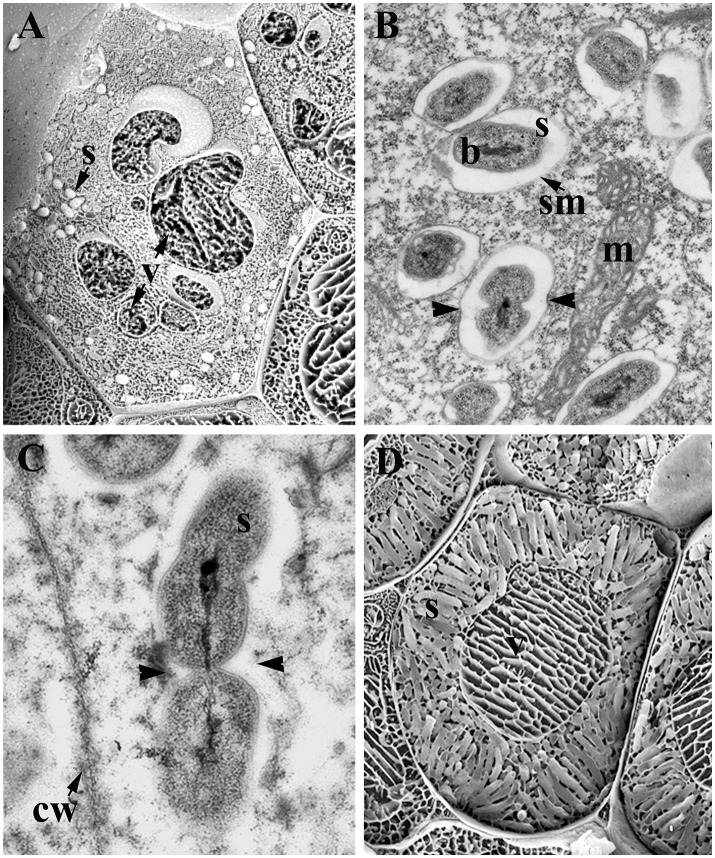
Infected cells of legume nodules. **(A)** Young infected cells showing few symbiosomes and vacuole disintegration; *Medicago sativa* nodule. **(B,C)** Symbiosomes in young infected cells; *M. sativa* **(B)** and *Lupinus albus* **(C)** nodules. Note the dividing symbiosomes (arrowheads). **(D)** Mature infected cells harboring mature symbiosomes; *M. sativa* nodule. Low temperature-scanning electron microscopy image **(A,D)**. Transmission electron microscopy image **(B,C)**. b, Bacteroid; cw, cell wall; m, mitochondria; s, symbiosome; sm, symbiosome membrane; v, vacuole.

Rhizobia can use intracellular or intercellular routes to infect legume roots. In the former, infection occurs at root hairs where infection threads (IT) form. IT grows inwardly until it reaches the nodule primordium cells. The intracellular mode of infection occurs in most of the rhizobia-legume symbioses studied and it is tightly controlled by the host. Intercellular infection may take place via natural wounds, where lateral roots emerge through epidermal breaks (crack infection), or it may occur directly between epidermal cells or between an epidermal cell and an adjacent root hair (Gualtieri and Bisseling, 2000; Vega-Hernández et al., 2001; González-Sama et al., 2004; reviewed in Sprent, 2009; and in Ibáñez et al., 2017). At least 25% of all legume genera may undergo non-hair rhizobia infection and their nodules lack ITs (Sprent, 2007). Rhizobia that enter the nodule host cell are surrounded by a host-derived membrane called the peribacteroid membrane or symbiosome membrane (SM). This new cellular compartment formed by the intracellular bacteria (bacteroid) enclosed within a SM is referred to as the symbiosome (Figure 1). Bacteria can divide within the symbiosome and whole symbiosomes can also divide inside the host cell, both these types of division being carried out synchronously or not (Whitehead and Day, 1997; Oke and Long, 1999). After rhizobia division ceases, the bacteria differentiate into nitrogen-fixing bacteroids. Plant defense reactions are suppressed or attenuated during the infection process (Mithöfer, 2002; Luo and Lu, 2014) or evaded (Saeki, 2011).

The symbiosome is the basic nitrogen-fixing unit of the nodule and the nitrogen fixed by bacteroids is exported as ammonium to the host plant cytoplasm, where it is assimilated and transported toward the rest of the plant. Conversely, reduced carbon compounds from the plant are transported to the nodule, and many other metabolites may also be exchanged between the host cell and symbiosome (Udvardi and Day, 1997; Hinde and Trautman, 2002). In 1997, it was first postulated that “symbiosomes can be interpreted as special nitrogen-fixing organelles within the host cell” (Whitehead and Day, 1997).

In most of the legumes studied, nodule host cells stop dividing upon rhizobia infection (Brewin, 1991), although young infected cells can still undergo cell division in several determinate nodules but this process is not sustained for long (Patriarca et al., 2004). Nevertheless, rhizobia-infected cell division does occur in some specific cases, such as the peculiar indeterminate nodule of *Lupinus* known as lupinoid nodule (González-Sama et al., 2004; Fedorova et al., 2007), and it is a key event in forming the infected tissue in which nitrogen will be fixed.

Here, we will present some evolutionary considerations regarding rhizobia-legume symbioses in general, and about *Lupinus* symbiosis in particular, leading us to suggest that different legume symbiosomes could represent some different stages in an evolutionary process toward a nitrogen-fixing organelle. First, we will introduce some evolutionary considerations about the origin of mitochondria and chloroplast, contrasting this with the apparent absence of diazotrophic organelles. We will compare the different degrees of association between diazotrophs and eukaryotes, and we will detail a number of evolutionarily relevant features of rhizobia-legume symbiosis. Finally, we will analyse the various organelle-like characteristics of the symbiosome, providing evidence suggesting that the symbiosome might be considered a nitrogen-fixing link in organelle evolution.

## The origin of mitochondria and chloroplasts as a model of organelle evolution. evolutionary considerations on the absence of nitrogen-fixing organelles

Biochemical, genetic, phylogenetic, and structural studies indicate that mitochondria are derived from an α-proteobacterium-like ancestor that was engulfed as a microsymbiont by an Archaea-type host between 2.2 and 1.5 Bya (Table 1; Dyall et al., 2004; Kutschera and Niklas, 2005; Gray, 2012). This specific symbiotic association was linked to the appearance of the first heterotrophic unicellular eukaryotes. Similarly, the primary origin of plastids is due to a symbiotic association between an ancient cyanobacterium and a mitochondrial carrying eukaryote, which took place between 1.5 and 1.2 Bya, giving rise to photosynthetic unicellular eukaryotes (Table 1; McFadden, 1999; Dyall et al., 2004; Kutschera and Niklas, 2005; Keeling, 2010).

**Table 1:** Cellular organelles derived from endosymbionts and putative connecting-link or intermediate stages in organelle evolution (adapted from Lang et al., 1997; Douglas and Raven, 2003; Kutschera and Niklas, 2005; Marin et al., 2005).

**Organelle**	**Ancestor**	**Age (million years ago)**	**Organelle function**	**Eukaryotic host**	**Morphological, physiological and molecular modifications**
Mitochondria	Ancient α-proteobacterium-like	2,200–1,500	Aerobic Respiration	Eukaryotes	High rates of gene loss (just 8-13 genes retained) Gene transfer to the host cell nucleus Protein import machinery Rapid sequence evolution No bacterial-like division
Atypical mitochondria	Ancient α-proteobacterium-like	More recent that typical mitochondria	Aerobic respiration	*Reclinomonas americana*	67 protein-encoding genes retained Eubacterial-like gene transcription Eubacterial-like protein sorting coexisting with evolving mitochondrial protein import machinery
Chloroplast	Ancient cyanobacterium-like	1,500–1,200	Photosynthesis	Photosynthetic eukaryotes	High rates of gene loss Gene transfer to the nucleus of the host cell Rapid sequence evolution Bacterial-like division
Chromatophores	Cyanobacterium-like plastids	200–60 More recent than typical chloroplast	Photosynthesis	*Paulinella chromatophora*	Reduced genome and gene transfer to the nucleus Protein targeting from the nucleus Division in synchrony with the host Peptidoglycan cell wall retained Similar pigmentation to many cyanobacteria Bacterial-like β-carboxysomes

The distinction between an endosymbiont and an organelle remains a matter of debate. It has been postulated that key aspects to distinguish an organelle from an endoysmbiont include the transfer of genes from the symbiont to the host nucleus, together with the establishment of a protein import apparatus in order to reimport the products of the transferred genes back into the compartment where they originally acted (Cavalier-Smith and Lee, 1985; Theissen and Martin, 2006; Keeling and Archibald, 2008; Archibald, 2015). Thus, a key event in the evolution from endosymbiont to organelle is the loss of autonomy of the microsymbiont as a free-living organism. This loss of autonomy is generally a consequence of microsymbiont genome reduction due to gene transfer to the host genome and gene loss (Dyall et al., 2004; Archibald, 2015). Such reduction is a continuous process (Douglas and Raven, 2003; Bock and Timmis, 2008) and the relocation of proto-organelle genes to the host genome may occur to avoid harboring duplicate sets of microsymbiont genes. Moreover, DNA transfer from organelles to the nucleus may drive gene and genome evolution (Kleine et al., 2009). An additional criterion thought to define an organelle is the host's control of organelle division and segregation (Keeling and Archibald, 2008). In the proposed major transitions approach, the evolution of symbiotic partnerships in the newly integrated organism is thought to be driven by the vertical transmission of symbionts into the host's offspring, a key event for the integration of both partners (Kiers and West, 2015).

Mitochondria and plastids, double membrane-surrounded cell organelles of endosymbiotic origin, fit with these criteria of reduced genome size, gene transfer to the host cell's nucleus, the presence of a protein import machinery, and host-driven division and segregation (Keeling, 2010; Strittmatter et al., 2010; Gray, 2012; Dudek et al., 2013). It is interesting to note that putative intermediate stages in mitochondrial and plastid evolution have been proposed. A heterotrophic flagellate of the genus *Reclinomonas* is reported to contain a minimally-derived mitochondrial genome with 67 protein encoding genes, many more than the mitochondrial genes conserved in yeast (8) and humans (13). Moreover, ancestral bacterial protein transport routes coexist with the evolving mitochondrial protein import machinery in *R. americana*. Accordingly, *Reclinomonas* mitochondria may represent a “connecting link” between the metazoan mitochondria and their ancestral bacterial progenitors (Lang et al., 1997, 1999; Tong et al., 2011).

The thecate amoeba *Paulinella chromatophora* contains obligate subcellular plastid-like photosynthetic bodies called chromatophores. It was estimated that these chromatophores evolved from free-living *Synechococcus* cyanobacteria 200–60 Mya (Nowack, 2014), although it is unclear whether these subcellular bodies should be considered as endosymbionts or organelles (Keeling and Archibald, 2008). Some years ago, the cyanobacterium-like plastids of the amoeba *P. chromatophora* were believed to represent intermediate forms in the transition from endosymbiont to plastids, these chromatophores retaining a prokaryotic peptidoglycan cell wall that is lost in current plastids (Keeling, 2004). These subcellular bodies have a smaller genome than their free-living relatives and they are metabolically dependent on their host. Indeed, several chromatophore genes have been transferred to the host nucleus and at least some of the proteins encoded by these genes are targeted to the chromatophores. Moreover, these subcellular bodies divide in synchrony with their host. Thus, in accordance to the aforementioned criteria, the chromatophores of *Paulinella* can be considered an early stage photosynthetic organelle that is the result of a relatively recent endosymbiotic event (Nowack et al., 2008; Nakayama and Ishida, 2009; Nakayama and Archibald, 2012; Nowack and Grossman, 2012; Archibald, 2015).

In contrast to mitochondria and chloroplasts, nitrogen-fixing organelles are absent in extant organisms, raising questions as to why these organelles have not yet appeared in the course of evolution (McKay and Navarro-González, 2002). Based on the close phylogenetic relationship between current diazotrophic bacteria (α-proteobacteria rhizobia and cyanobacteria) and the most likely free-living ancestors of mitochondria or chloroplasts, there doesn't appear to be any fundamental incompatibility of diazotrophic predecessors for endosymbiosis and for the transfer of nitrogen-fixing genes to the host cell's nucleus (Allen and Raven, 1996).

Nitrogenase is inhibited by oxygen, so nitrogen-fixing organisms might have appeared before the Great Oxidation Event more than 2 Bya (i.e., the accumulation of oxygen in the atmosphere) (Raymond et al., 2004). Nitrogen-fixing organisms probably originated in a time when there was a shortage in the availability of fixed-nitrogen. Three nitrogen crises have been proposed during evolution: the first just after the origin of life (more than 3.5 Bya); the second possibly due to a strong reduction in atmospheric CO_2_ (about 2.5 Bya); and the third, possibly induced by the action of pluricellular plant-based ecosystems (500 Mya; McKay and Navarro-González, 2002).

Isotope studies suggest that biological nitrogen fixation first took place about 3.2 Bya (Stüeken et al., 2015). It was also postulated that biological nitrogen fixation appeared later than the genesis of the eukaryotic cell (McKay and Navarro-González, 2002, and references therein) and molecular dating suggested that the origin of biological nitrogen fixation was between 2.2 and 1.5 Bya (Fani et al., 2000; Boyd and Peters, 2013). Thus, biological nitrogen fixation could have appeared during the second nitrogen crisis. Eukaryogenesis had been completed by then and it was a single event. Thus, for whatever reason, an opportunity for new endosymbiosis between the unicellular eukaryotic cell and diazotrophs did not arise. If nitrogen-fixing organisms appeared during the third crisis, higher plants already existed, and thus, incorporation and vertical transmission in multicellular organisms was much more difficult (McKay and Navarro-González, 2002). Indeed, it has been postulated that organelle development does not occur in differentiated multicellular organisms (McKay and Navarro-González, 2002).

A very interesting case of co-evolution involving a permanent nitrogen-fixing endosymbiont can be found in diatoms of the Rhopalodiaceae family, protists that contain the so-called spheroid bodies (SB) in their cytoplasm. As in the case of rhizobia-legume symbioses, the host and microsymbiont are strictly separated by a host-derived membrane in these species (Drum and Pankratz, 1965; Prechtl et al., 2004; Bothe et al., 2010). Moreover, phylogenetic analyses showed that these SBs are derived from a group of cyanobacteria and that their genome is closely related to that of nitrogen-fixing bacteria of the genus *Cyanothece* (Adler et al., 2014). This is a case of obligate symbiosis with vertical transmission, because SBs cannot survive outside the host cells (Prechtl et al., 2004). Indeed, this seems to be a case of recent symbiosis induced by a loss of photosynthetic capacity of the cyanobacteria-derived symbiont (Prechtl et al., 2004). This endosymbiosis was proposed to have occurred in the middle Miocene epoch, ~12 Mya (Nakayama et al., 2011). The complete genome of a SB from one of these diatom species was recently sequenced (Nakayama et al., 2014), confirming the reduced size and gene repertoire of the SB relative to their closer free-living relatives. Furthermore, the presence of pseudogenes and gene fusions suggest an ongoing process of genome reduction. Interestingly, the genome of SBs contains a set of genes for nitrogen fixation and isotope analysis indicated that the host diatoms use the nitrogen fixed by the SBs (Nakayama and Inagaki, 2014). However, genes for functional photosynthesis are lacking in its genome and thus, SBs depend on their diatom hosts for their energy requirements. To date, SBs have not been considered as organelles *stricto sensu*, as gene transfer to the host nucleus and protein import machinery have not yet been detected. Moreover, little is known about endosymbiont division and segregation to host daughter cells (Adler et al., 2014; Nakayama and Inagaki, 2014).

It is interesting to note that some unicellular nitrogen-fixing cyanobacteria of the oceanic picoplankton, termed UCYN-A, have suffered a more pronounced reduction of their genome than that observed in SBs. These cyanobacteria lack genes that code for several metabolic pathways, yet they are evolutionarily related to SBs. It has been proposed that these cyanobacteria may enter into symbiosis with prymnesiophyte photosynthetic unicellular algae, supplying fixed nitrogen to the host and receiving fixed carbon in return (Thompson et al., 2012; Nakayama and Inagaki, 2014). Like SBs, this relationship between UCYN-A cyanobacteria and unicellular algae can be considered another stage in the evolution of symbiosis involving nitrogen-fixation.

## Different levels of interaction between diazotroph bacteria and eukaryotes

Only some diazotroph bacteria are known to establish symbiotic interactions with eukaryotes, be they animal, plant, fungus, or protist. These interactions range from loose associations to highly specific intracellular symbioses, involving different molecular, physiological, and morphological modifications. As such, the co-evolutionary status of these associations can be estimated by considering the degree of interdependence (facultative or obligate symbiont), the extra- or intracellular location of the microsymbiont, the presence or absence of segregation to daughter cells and of vertical transmission (Kneip et al., 2007). Some examples of diversity of interactions between diazotroph and plants or photosynthetic protists are shown in Table 2.

**Table 2:** Some associations of diazotrophs with photosynthetic eukaryotes.

**Diazotroph**	**Eukaryotic host**	**Microsymbiont location**	**Degree of dependence**	**Infection mode**	**Host niche**	**Infected cell division**	**Vertical transmission**	**Characteristic features of bacteria**	**References**
*Azospirillum* sp. *Azoarcus* sp.	Poaceae *Zea mays Oryza sativa*	Extracellular	Facultative	None	Intercellular spaces in roots and other plant tissues No nodules	No	No	None	Hurek et al., 1994 Reinhold-Hurek and Hurek, 1998, 2011 Steenhoudt and Vanderleyden, 2000
*Nostoc* sp.	Bryophyta *Anthoceros punctatus*	Extracellular	Facultative	None	Cavities of the gametophyte No nodules	No	No	Increased heterocyst frequency	Endelin and Meeks, 1983 Adams and Duggan, 2008
*Nostoc* sp. or *Anabaena* sp.	Monilophyta *Azolla* sp.	Extracellular	Obligate	Infection of sexual megaspore	Cavities in the dorsal leaf that are obligately infected by filamentous cyanobacteria No nodules	No	Yes	Cyanobiont genome degradation	Bergman et al., 2008
*Nostoc* sp. (predominant)	Cycads *Encephalartos* sp. *Macrozamia* sp. Others	Extracellular	Facultative	Infection of coralloid roots (somewhat comparable to crack entry)	Coralloid roots No nodules	No	No	Irreversibly modified coralloid roots	Rasmussen and Nilsson, 2002 Vessey et al., 2004
*Nostoc* sp.	Angiosperm *Gunnera* L.	Intracellular	Facultative	Plant stem glands	Specialized plant stem glands No nodules	No	No	Differentiation of *Nostoc* filaments	Rasmussen et al., 1994 Bergman et al., 2008
*Frankia* sp.	Dicotyledonous actinorrhizal plants Casuarinaceae and others (*Alnus* sp., *Casuarina* sp., etc.)	Intracellular	Facultative	Infection thread –like structures or penetration between root epidermal cells	Symbiotic nodule Indeterminate Multi-lobed Central vasculature	No	No		Miller and Baker, 1986 Vessey et al., 2004 Pawlowski and Sprent, 2008 Kucho et al., 2010
*Frankia* sp. of cluster II	Actinorhizal Rosales and Cucurbitales	Intracellular	Likely obligate	Unknown	Symbiotic nodule Indeterminate Multi-lobed	No	Unknown	High percentage of pseudogenes Proposed genome reduction	Pawlowski and Sprent, 2008 Persson et al., 2011
*Bradyrhizobium* sp. *Rhizobium* sp.	Cannabaceae (*Parasponia* sp.)	Intracellular	Facultative	Crack entry Root erosion (Intercellular infection-thread)	Symbiotic nodule Indeterminate Central vasculature (similar to actinorrhizal nodules)	No	No	Fixation threads	Trinick, 1973 Becking, 1979 Vessey et al., 2004
*Ensifer* sp. *Rhizobium* sp.	*Medicago* sp. *Pisum* sp. *Vicia* sp.	Intracellular	Facultative	Root hair Infection thread	Symbiotic nodule Indeterminate. Cylindrical Peripheral vasculature	No	No	Irreversible differentiation into bacteroids	Newcomb, 1981 Rae et al., 1992
*Mesorhizobium* sp. *Rhizobium* sp.	*Lotus* sp. *Phaseolus* sp.	Intracellular	Facultative	Root hair Infection thread	Symbiotic nodule Determinate	Limited	No		Newcomb, 1981 Pankhurst et al., 1987
*Bradyrhizobium* sp.	*Chamaecytisus proliferus*	Intracellular	Facultative	Crack entry	Symbiotic nodule Indeterminate. Cylindrical	Limited	No	Aborted infection threads	Vega-Hernández et al., 2001
*Bradyrhizobium* sp.	*Glycine max*	Intracellular	Facultative	Root hair Infection thread	Symbiotic nodule Determinate	Limited	No		Bhuvaneswari et al., 1980
*Bradyrhizobium* sp.	Dalbergioids *Arachis hipogea Stylosanthes* sp. *Sarothamnus scoparius*	Intracellular	Facultative	Crack entry	Symbiotic nodule Determinate Aeschynomenoid	Yes	No		Chandler, 1978 Chandler et al., 1982 Sajnaga et al., 2001 Saeki, 2011
*Bradyrhizobium* sp.	Genistoid *Genista tinctorea*.	Intracellular	Facultative	Intercellular	Symbiotic nodule Indeterminate	Yes	No		Kalita et al., 2006
*Bradyrhizobium* sp.	Genistoid *Lupinus* sp.	Intracellular	Facultative	Intercellular	Symbiotic nodule Indeterminate lupinoid	Yes	No		Tang et al., 1993 Lotocka et al., 2000 González-Sama et al., 2004 Fedorova et al., 2007
*Cyanothece* sp.	Diatom *Rhopalodia gibba*	Intracellular	Obligate	Obligate endosymbiont	Spheroid bodies No nodules	Yes	Yes	Loss of genes Accumulation of deleterious mutations	Drum and Pankratz, 1965 Kneip et al., 2008 Bothe et al., 2010

Examining these diazotroph-plant interactions has enabled different degrees of specialization to be defined. For example, *Azospirillum* sp., *Azoarcus* sp., and some other free-living diazotroph bacteria are plant-growth promoting bacteria that can establish interactions with different cereals by root colonization or endophytic association, and they profit from microaerobic environments to fix nitrogen while obtaining nutrients from the plant's roots (Reinhold-Hurek and Hurek, 1998, 2011; Steenhoudt and Vanderleyden, 2000; Pérez-Montaño et al., 2014). Another example of a relative loose association involving diazotrophs is the symbiosis established between the cyanobacteria *Nostoc* sp. and the bryophyte *Anthoceros punctatus* L. (Adams and Duggan, 2008). In this case, the microsymbiont is located extracellularly in the cavities of the gametophyte and one physiological adaptation of this is that the heterocyst frequency in *Nostoc* sp. is higher than in free-living conditions (Endelin and Meeks, 1983).

In the symbiosis between cyanobacteria (*Nostoc* or *Anabaena*) and the fern *Azolla*, the diazotroph microsymbiont resides extracellularly in a mucilaginous sheath in the dorsal cavities of *Azolla* leaves. The cyanobacteria's filaments enter into the fern's sexual megaspore, allowing the microsymbiont to be transferred vertically to the next plant generation. While it retains its photosynthetic capacity, it seems that these diazotroph cyanobacteria have lost their capacity to survive as free-living organisms (Bergman et al., 2008). Indeed, there are signs of reductive genome evolution or degradation of the cyanobiont, i.e., the presence of a high proportion of pseudogenes and a high frequency of transposable elements (Larsson, 2011). As such, it has been proposed that this cyanobiont may be at the initial phase of the transition from a free-living organism to a nitrogen-fixing plant entity, similar to chloroplast evolution (Ran et al., 2010). Moreover, it is possible that this *Nostoc* symbiosis may have persisted for 200 million years (Bergman et al., 2008).

All gymnosperm cycads can establish root symbioses with *Nostoc* sp. and with other cyanobacteria (Thajuddin et al., 2010). Cyanobacteria invade a particular root type, the cycad coralloid roots, provoking irreversible morphological modifications. The cyanobacteria remain extracellular in this symbiosis, which could have originated up to 250 Mya (Vessey et al., 2004 and references therein). A different strategy is adopted in the symbiosis between *Nostoc* sp. and the angiosperm *Gunnera* L. These bacteria infect specialized plant stem glands to become intracellular. Indeed, these glands secrete a specific signaling molecule that induces the differentiation of *Nostoc* filaments into a specialized form that is essential for infection (Rasmussen et al., 1994; Bergman et al., 2008). Moreover, *Nostoc* filaments are always surrounded by a host plasma membrane. In these examples, cyanobacteria fix nitrogen in both free-living and symbiotic conditions, and symbiosis is facultative and there has been no vertical transmission observed (Bonnett and Silvester, 1981; Rasmussen et al., 1994; Santi et al., 2013).

Root-nodule symbioses can be established between higher plants and soil bacteria, and it was estimated that nitrogen-fixing root nodule symbioses evolved 50–100 Mya (Kistner and Parniske, 2002). Symbiosis of the actinorhiza *Frankia* originated about 70–90 Mya (Doyle, 1998, 2011; Hocher et al., 2011), while legume-rhizobia symbiosis originated about 55–60 Mya (Lavin et al., 2005), *Parasponia*-rhizobia symbiosis is much more recent (less than 10 million years; Op den Camp et al., 2011). In actinorhizal symbioses, soil actinobacteria of the genus *Frankia* induce nodules in the roots of about 260 plant species from eight different families of dicotyledonous plants (Vessey et al., 2004; Benson and Dawson, 2007). *Frankia* can fix nitrogen as a free-living organism and it can enter the host plant root either intracellularly (through root hairs) or intercellularly, depending on the host plant species. *Frankia* induces the formation of multi-lobed, indeterminate nodules, which are modified adventitious secondary roots formed from the root pericycle. Nodule infected cells become full of branching *Frankia* hyphae surrounded by a perimicrobial membrane of host origin, forming vesicles in which nitrogen fixation takes place (Vessey et al., 2004; Pawlowski and Sprent, 2008; Kucho et al., 2010; Froussart et al., 2016). This symbiosis is usually facultative but *Frankia* strains of cluster II, which form symbiosis with actinorhizal Rosales and Cucurbitales, still cannot be cultured and thus, these actinobacteria are probably obligate symbionts (Pawlowski and Sprent, 2008). The failure to culture these microbial strains may be related with atypical patterns of auxotrophy (Gtari et al., 2015). The genome of a member of this cluster is small and with a relatively high proportion of pseudogenes, suggesting that this strain underwent a process of genome reduction and that genome degradation is ongoing (Persson et al., 2011). However, this genome reduction does not involve physiological impairment, as no metabolic pathways appear to be incomplete. Notably, it also contains fewer genes involved in stress responses.

**Figure 2:**
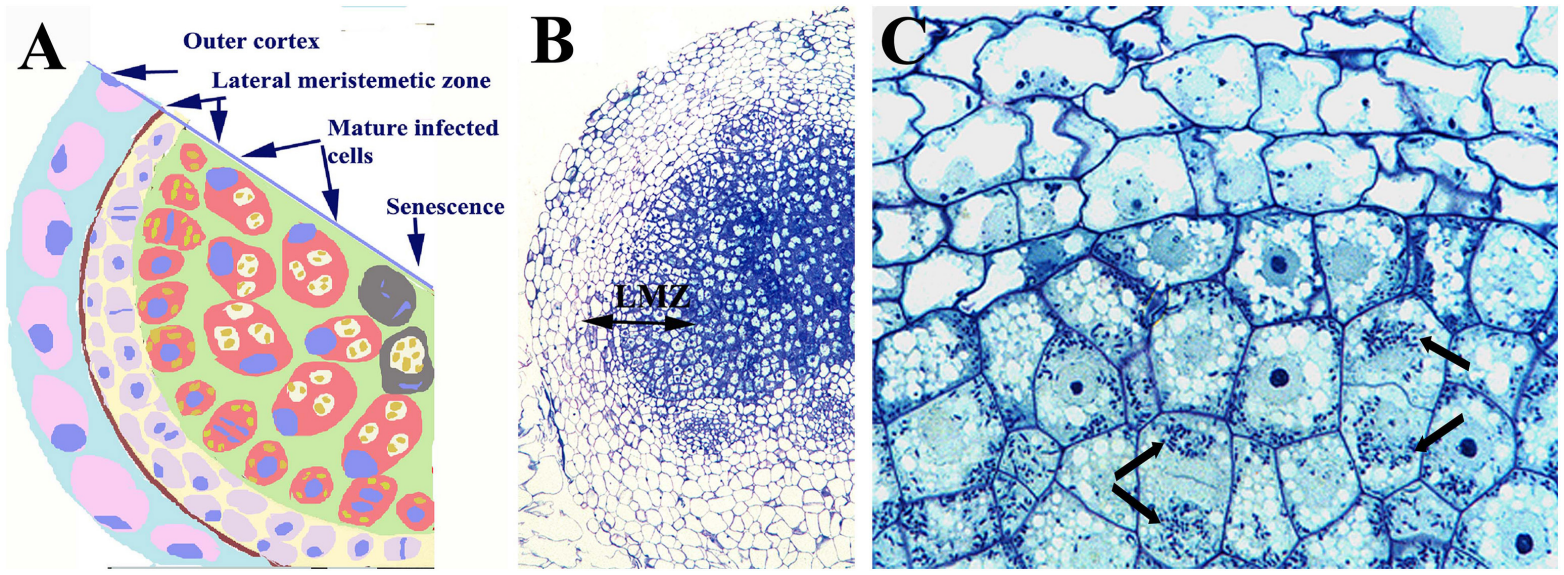
Nodule of *Lupinus albus* showing dividing infected cells. **(A)** Scheme of a nodule section and **(B)** light microscopy image showing the outer cortex, and the lateral meristematic zone (LMZ) composed of infected and uninfected dividing cells, as well as the central zone composed of infected cells. **(C)** Detail of the LZM in which the arrows label the symbiosomes. Note the symmetric distribution of symbiosomes between daughter cells. Images **(B,C)** modified from Fedorova et al. (2005); they are being reproduced with permission from the copyright holder.

**Figure 3:**
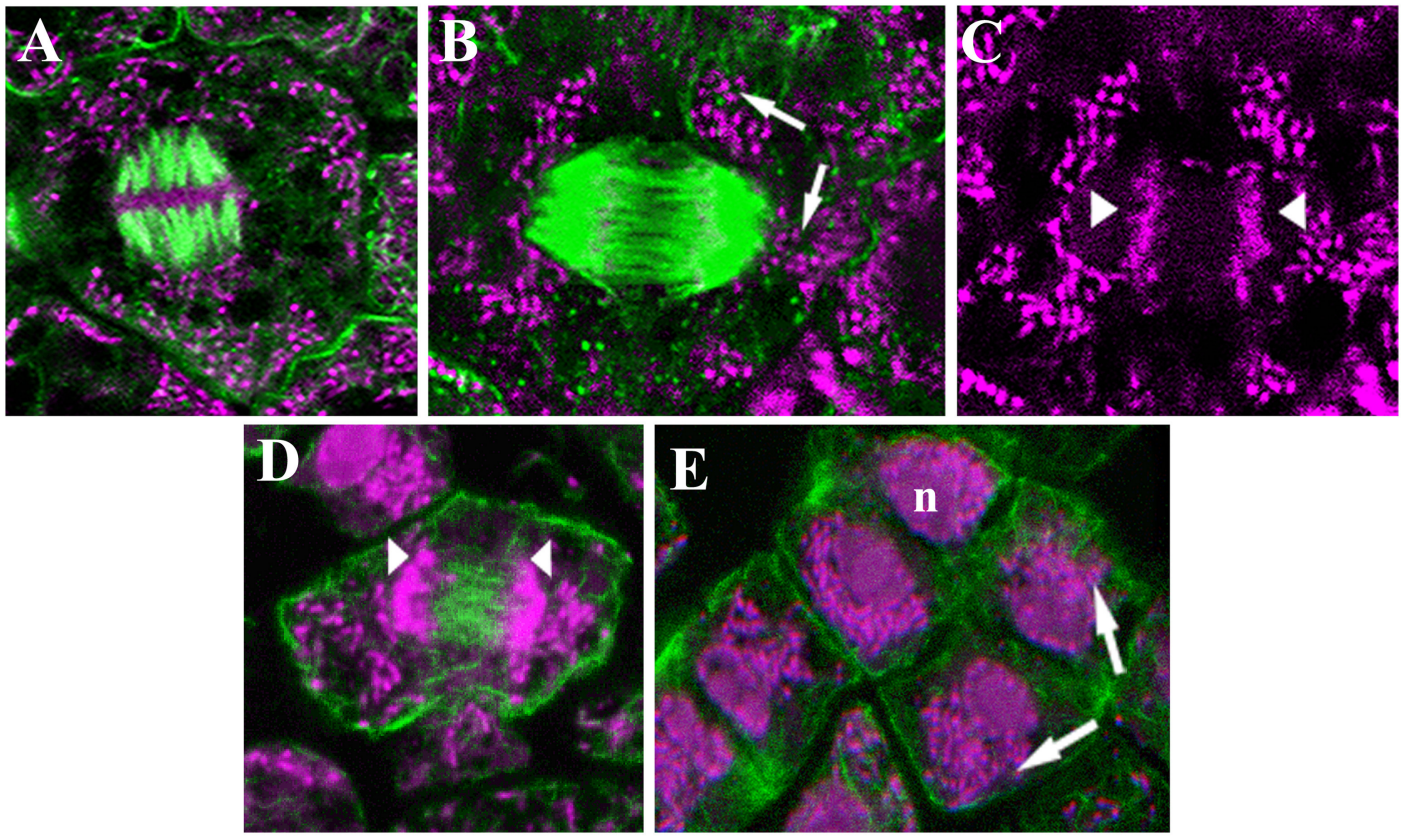
Confocal laser images of dividing infected cells of *Lupinus albus* nodules showing the cytoskeletal elements in green, and the DNA of bacteroids (arrow) and chromosomes (arrowhead) in magenta. **(A–D)** Metaphase, microtubules **(A,B)** and actin microfilaments **(D)**. **(E)** Different anaphase–telophase stages showing the actin microfilaments. n, Nuclei. Images modified from Fedorova et al. (2007); they are being reproduced with permission from the copyright holder.

The symbiosis established between rhizobia and legumes is very specific and it involves a more complex exchange of signals and the development of a root nodule. This structure is not a modified root (as in the case in cycads, actinorhizal plants and *Parasponia*) but rather, it arises from unique zones of cell division in the root cortex (Vessey et al., 2004). Most rhizobia can only fix nitrogen in symbiotic conditions, when the bacteria have differentiated into bacteroids (the nitrogen-fixing form) inside the symbiosomes within the nodule's host cells (Brewin, 1991; Whitehead and Day, 1997). In most symbioses, legume host cells do not further divide once infected by the bacteria. This is the case for thread-infected indeterminate nodules formed by *Pisum* or *Medicago*. It has been suggested that young cells in thread-infected determinate nodules, such as those formed by *Glycine, Lotus*, or *Phaseolus*, undergo cell division but not in a sustained manner (Patriarca et al., 2004). In the case of the symbiosis established between *Bradyrhizobium* and *Arachis* or *Stylosantes*, giving rise to determinate nodules, infected cells can divide (Chandler, 1978; Chandler et al., 1982). In lupinoid nodules formed by *Lupinus albus*, infected host cells continue to divide for several cycles (Fedorova et al., 2007) and indeed, the lupinoid nodule grows continually and maintains an active lateral meristem with infected dividing cells (Figure 2), allowing the segregation of symbiosomes between daughter cells (Figure 3). Nevertheless, legume symbiosis is facultative and no vertical transmission occurs, such that new infection by rhizobia must occur for each new plant generation and no gene transfer from micro- to macro-symbiont has been reported.

*Parasponia* (Cannabaceae, order Rosales) is the only non-legume plant that can establish effective nodule symbiosis with rhizobia. This symbiosis is a case of convergent evolution and it occurred more recently than that of legumes. From a phylogenetic and taxonomic point of view, *Parasponia* is closer to some actinorhizal plants that belong to the Rhamnaceae, Elaeagnaceae, and Rosaceae families, than to legumes (Soltis et al., 1995; Geurts et al., 2012). *Parasponia* nodules are modified lateral roots that originate from the pericycle, and they are indeterminate and more similar to actinorhizal nodules than to legume nodules. The entry of symbiotic bacteria (*Rhizobium, Bradyrhizobium*) does not involve root hairs but rather, crack entry or root erosion and an intercellular IT. This IT protrudes into the host plant cell by plant membrane invagination, forming the so-called fixation thread. Fixation-thread, that remains in contact with the plasma membrane, are the equivalent to a symbiosome in legumes and to arbuscules in arbuscular mycorrhizal (AM) roots (Vessey et al., 2004; Pawlowski and Sprent, 2008; Behm et al., 2014). AM symbiosis preceded root nodule symbioses and the interactions of plants with AM fungi probably originated more than 400 Mya (Bonfante and Genre, 2008). This symbiosis is wide spread, involving more than 80% of all terrestrial plants, and fungi from order Glomales (Harrier, 2001). In this symbiosis, AM fungi enter the roots and spread into the inner cortex by invagination of the plasma membrane. Invading hyphae branch and they develop the arbuscule, a specialized structure that is subsequently enveloped by the periarbuscular membrane, an extension of the host plant's plasma membrane. A symbiotic interface between the arbuscule and the periarbuscular membrane controls the efficient exchange of nutrients between both symbionts, including the transfer of phosphorus and nitrogen from the fungus in return for photosynthates from the plant (Smith and Read, 2008). It is notable that some components of the signaling pathway required to establish rhizobia-legume symbiosis and the symbiotic interface are also present in AM symbiosis (Kouchi et al., 2010; Harrison and Ivanov, 2017).

As described above, the endosymbiosis of SBs related to the cyanobacterium *Cyanothece* sp., with the diatom *Rhopalodia gibba* and some other species, seems to be a unique case of obligate nitrogen-fixing endosymbiosis, involving genome reduction, a lack of metabolically essential genes and vertical transmission. As indicated above, the microsymbiont is currently not considered a real organelle due to the lack of gene transfer to the host nucleus and of a protein import machinery (Nakayama and Inagaki, 2014).

## Evolutionary considerations about individual symbionts in rhizobia-legume symbioses

### Some genetic and evolutionary characteristics of the microsymbiont

In general terms, rhizobia are defined as soil bacteria that fix nitrogen in symbiotic association with legumes and *Parasponia*. The Proteobacteria is an important phylum that contains diazotrophic organisms and phylogenetic studies using 16S ribosomal RNA sequences indicate that the best-known rhizobial genera are from the α-proteobacteria group (Rogel et al., 2011; Weir, 2016), including the genera: *Rhizobium, Mesorhizobium, Sinorhizobium* (renamed *Ensifer*, Martens et al., 2007; Judicial Commission of the International Committee on Systematics of Prokaryotes, 2008), *Bradyrhizobium, Azorhizobium*, and *Allorhizobium*. Some other α-proteobacteria genera also contain one or more rhizobial species, such as *Aminobacter, Methylobacterium, Devosia, Ochrobactrum, Phyllobacterium, Microvirga*, and *Shinella* (Rogel et al., 2011; Ormeño-Orrillo et al., 2015; Weir, 2016; ICSP Subcommittee on the taxonomy of *Rhizobium* and *Agrobacterium* http://edzna.ccg.unam.mx/rhizobial-taxonomy/). Recently, *Neorhizobium* and *Pararhizobium* have been proposed as new genera (Mousavi et al., 2014, 2015). Several rhizobial species belong to the β-proteobacteria genera, including *Burkholderia, Cupriavidus*, and *Herbaspirillum* (Moulin et al., 2001; Chen et al., 2003; Lloret and Martínez-Romero, 2005; Masson-Boivin et al., 2009; Rogel et al., 2011; Weir, 2016). Indeed, the taxonomy of rhizobia has recently been revised (Peix et al., 2015; Shamseldin et al., 2017).

In a first instance, a comparison of glutamine synthetase (GS) genes I and II allowed the time of divergence among the α-proteobacteria genera of rhizobia to be estimated (Turner and Young, 2000). The data from GSII sequences suggest that *Rhizobium* and *Ensifer* are the most recent genera, and *Bradyrhizobium* and *Mesorhizobium* the most ancient. Based on GSI, *Rhizobium, Ensifer*, and *Mesorhizobium* genera appear to have separated at the same time, and *Bradyrhizobium* is the most ancient genus. Based on the analysis of GS genes and the amino acid substitution rates in their orthologs, the *Bradyrhizobium* genus probably diverged from the last common ancestor of all rhizobia some 500 Mya, before the appearance of land plants (about 400 Mya). Similarly, the most recent genus *Ensifer* diverged about 200 Mya (Turner and Young, 2000; Morton, 2002; Lloret and Martínez-Romero, 2005), before the appearance of Angiosperms (dated more than 150 Mya; Martin et al., 1989) and legumes (about 70 Mya; Lavin et al., 2005). When phylogenetic analyses of the 16S rRNA gene and the intergenic spacer region was combined, slightly but not significantly more recent divergence times were found for rhizobia: about 385 Mya for *Bradyrhizobium*, 344 Mya for *Mesorhizobium*, 201 Mya for *Ensifer*, 145 Mya for *Rhizobium/Agrobacterium*, and 54 Mya for *Neorhizobium* (Chriki-Adeeb and Chriki, 2016).

Evolutionary studies of α-proteobacteria indicate that while the evolution of a reductive genome has been observed in intracellular animal-associated bacteria, genome expansion is observed in plant symbionts (as well as in several animal and plant pathogens, such as *Rickettsia, Brucella*, or *Bartonella*). Rhizobia are among the α-proteobacteria with the largest genomes (MacLean et al., 2007) and genes involved in nitrogen fixation and nodulation (or pathogenicity) have become integrated for symbiosis, often arranged on auxiliary replicons in genomic islands (mobile elements). The genome size and the diversity among rhizobia are due to the presence of these highly dynamic auxiliary replicons and to a high degree of paralogy (Batut et al., 2004).

Genome plasticity and instability in rhizobia is due to largescale recombination events (the presence of repeated DNA sequences, insertion elements and multiple replicons), and in fact, lateral gene transfer is the primary source of genetic diversity in rhizobia (Flores et al., 2000; Guo et al., 2003; MacLean et al., 2007; Provorov et al., 2008). It has been proposed that the genomes of rhizobia have evolved by expansion as a means to adjust to the challenges imposed by their multiphase lifestyle, principally through horizontal gene transfer and gene duplication (Batut et al., 2004; MacLean et al., 2007; Provorov and Andronov, 2016). In some rhizobia-legume symbioses, up to 15-20% of the rhizobial genome is activated in symbiosis (Udvardi et al., 2004; Tikhonovich and Provorov, 2009). Different models of co-evolution in the rhizobia-legume symbiosis have been proposed or are under study (but they are still controversial); especially in relation to the selection of rhizobial symbiotic traits by the host legume (Provorov et al., 2008; Martínez-Romero, 2009).

An evolutionary step from free-living diazotrophs related to *Rhodopseudomonas* to the symbiotic diazotroph *Bradyrhizobium* through the acquisition of *fix* genes was proposed as the first stage of rhizobial evolution (Provorov, 2015; Provorov and Andronov, 2016). It is noteworthy that when compared to other well-known rhizobia, *Bradyrhizobium* displays several particular genomic and physiological characteristics related to diazotrophy and symbiosis. For example:

- *Bradyrhizobium japonicum* strains have some of the largest bacterial chromosomes sequenced to date (9.1–9.6 Mb), and the largest of all rhizobia (Kündig et al., 1993; Kaneko et al., 2002, 2011; Batut et al., 2004; Siqueira et al., 2014).
- The nodulation genes in *Bradyrhizobium* are located in a chromosomal segment that could be a mobile element, whereas they are located in symbiotic plasmids in other genera such as *Rhizobium, Ensifer*, and several strains of *Mesorhizobium* (Minamisawa et al., 1998; Sessitsch et al., 2002).
- Some *Bradyrhizobium* strains do not have genes for Nod factors but they can induce nodulation in certain some legumes of the *Aeschynomene* genus using an alternative triggering molecule (Giraud et al., 2007). This is a unique case of nodulation by rhizobia that does not involve Nod factors.
- *Bradyrhizobium* is the only genus of rhizobia in which some species (those nodulating some species of the *Aeschynomene* genus by a Nod factor-independent mechanism) can perform photosynthesis and fix nitrogen in symbiosis or in free-living conditions (Molouba et al., 1999). Other rhizobia can only fix nitrogen in symbiotic conditions, with the exception of *Azorhizobium caulinodans* and some strains of *Burkolderia* (Sprent et al., 2017).
- *Bradyrhizobium* displays an atypical two-component regulatory system, NodV and NodW, which is involved in controlling *nod* gene expression (Stacey, 1995; Loh et al., 1997) and in activating type III secretion system (Deakin and Broughton, 2009).
- In certain legumes belonging to the dalbergioid and genistoid genera, *Bradyrhizobium* induces nodules in which the host cells divide for several cycles after infection (Vega-Hernández et al., 2001; González-Sama et al., 2004; Kalita et al., 2006; Fedorova et al., 2007). This peculiarity makes the symbiosome of nodules formed by *Bradyrhizobium* in these legumes a prominent candidate in the evolutionary pathway toward “genetically obligatory symbiosis.”

### Some evolutionary and phylogenetic considerations about the macrosymbiont

All angiosperms that perform symbiotic nitrogen-fixing symbioses (except *Gunnera*) are included in the Rosid I clade (Soltis et al., 2000). This clade includes actinorhizal plants and plants that are nodulated by rhizobial bacteria. Recent phylogenetic and molecular data suggest that these nitrogen-fixing plants are derived from a common ancestor of the Rosid I clade with a genetic predisposition for nodulation (Soltis et al., 1995; Pawlowski and Sprent, 2008; Hocher et al., 2011). It was proposed that rhizobial symbioses has evolved four times independently within the Rosid I clade, three times for legumes and once for *Parasponia* (Doyle, 1998; Pawlowski and Sprent, 2008; Sprent, 2008). More recently, it was postulated that there might have been six to seven separate origins of nodulation in legumes (Doyle, 2011).

All plants nodulated by rhizobia are included in the family Leguminosae, except *Parasponia*. Leguminosae comprises more than 700 genera and about 20,000 species (Doyle, 2011), divided into three subfamilies: Caesalpinioideae, Mimosoideae, and Papilionoideae, although the legume taxonomy is currently under revision (Sprent et al., 2017). A key evolutionary study of the Leguminosae family has been performed taking into account molecular and fossil data (Lavin et al., 2005), concluding that legumes evolved about 60 Mya. It was postulated that nodulation could have developed due to an important climatic change at that time, involving an important increase in CO_2_ levels that made nitrogen limiting for plant growth (Sprent, 2007). A crucial first step in rhizobia-legume symbiosis is the capacity for mutual recognition and it is thought that this capacity derived from ancient arbuscular mycorrhizal symbiosis (Szczyglowski and Amyot, 2003). In fact, arbuscular mycorrhizal fungi secrete soluble LCO signals (Gough and Cullimore, 2011; Maillet et al., 2011) that are essential for arbuscular mycrorrhiza development in legumes, indicating there is a common signaling pathway for both rhizobia-legume and arbuscular mycorrhizal symbioses (Capoen et al., 2009; Markmann and Parniske, 2009; Genre and Russo, 2016).

**Figure 4:**
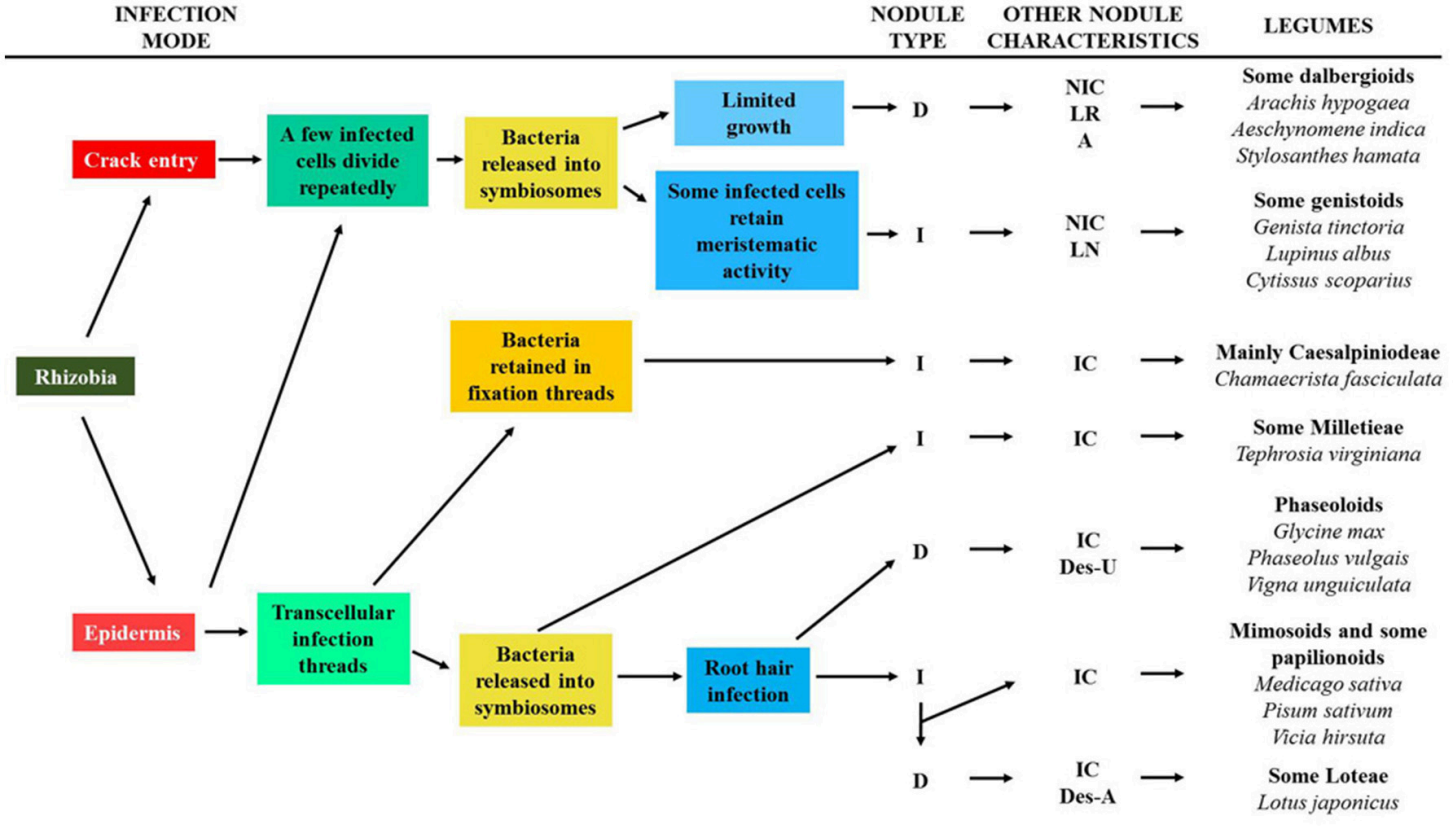
Scheme for the evolution of different legume nodules and major steps in the nodulation. The characteristics of some legume clades regarding nodulation are shown. D (determinate nodule); I (indeterminate nodule); IC (nodule containing interstitial cells); NIC (nodule lacking interstitial cells); LR (nodule associated with lateral roots); A (aeschynomenoid nodule); LN (lupinoid nodule, only for *Lupinus*); Des-U (desmodoid nodule exporting ureide); Des-A (desmodoid nodule exporting amide). It was adapted from Sprent and James (2007) and Oono et al. (2010).

The macrosymbiont determines the mode of root infection by rhizobia, and the structure and morphology of the nodule. The way of infection has been related to the evolution of legume nodulation, while the structure and morphology of nodules are different among legume clades and may be markers of legume phylogeny (Sprent, 2007, 2009; Sprent et al., 2013). It was considered that the infection processes and nodule structure are more important taxonomic characteristics of legumes than their ability or inability for nodulation (Sprent et al., 2017). An evolutionary scheme of the different rhizobia infection types and the nodule structure of extant legumes has been proposed (for details of this scheme and for examples of the legume nodules on which the model is based see: Sprent, 2007, 2008, 2009; Sprent and James, 2007; and Ibáñez et al., 2017). In this scheme the origin of rhizobia infection could either be through direct epidermal infection or crack infection, which would produce two distinct branches of nodule evolution (Figure 4). The more complex evolutionary line involves the formation of transcellular ITs and their entry into some daughter cells of the meristem. In a further evolutionary step, bacteria could be retained in a modified IT (no bacteria released into the host cell and consequently, no symbiosome is formed), as observed in Caesalpinioideae and Papilionoidae legumes. Alternatively, bacteria could be released into the host cell to form the symbiosome. The infection of root hair would be a later, key event in the evolution of the determinate and indeterminate nodules found in Mimosoidae, and in some Papilionoidae and Loteae legumes. All nodules originated in this evolutionary line contain infected and uninfected cells in their nitrogen-fixing zone. About 75% of nodulated legumes, including almost all mimosoids and Caesalpiniodeae, and more than 50% of papilionoids, would have followed this strategy.

In the other branch of nodule evolution a few cells are infected by rhizobia and they divide repeatedly (Figure 4). The bacteria enter the host cytoplasm in symbiosomes but not via an IT because no such structure is formed. The most distinctive structural feature of these nodules is that the infected zone is composed of only infected cells. Nodules evolved in this way are only found in Papilionoidae legumes and they include the determinate dalbergoid nodules (crack infection, aeschynomenoid nodules), and those of many Genistae and some Crotalarieae legumes (epidermal infection and some infected cells with meristematic activity: indeterminate nodules and lupinoid nodules).

The Papilionoid crown node arose about 58 Mya, while the genistoid and dalbergioid nodes date to about 56 and 55 Mya, respectively. In comparison, galegoid legumes (a clade that includes *Medicago, Vicia*, and *Pisum*) began their spread about 39 Mya and thus, it is the genistoid and dalbergioid that have the oldest origin within the papilionoids (Sprent, 2007; Hane et al., 2017). All legumes that originated later than 40 Mya form their nodules by root hair infection (Sprent, 2009).

In the framework of this review, it is interesting to note some features of the genistoid legume lupin. The *Lupinus* genus includes about 300 species that can be found all over the world. Although they predominantly exist on the American continent and in the Mediterranean area, some Mediterranean species have been introduced into Australia and South Africa. *Lupin* species colonize different environments and they have particular agronomic potential as they are more tolerant to certain abiotic stresses than other legumes (Fernández-Pascual et al., 2007). These legumes can grow in nitrogen and phosphate depleted soils, and their capability to exploit poor, degraded, contaminated or stress-affected soils, and produce safe, protein-rich seeds make *Lupinus* a legume of great interest (Lucas et al., 2015). The *Lupinus* genus has the fastest evolution rate in plants and species from the Andes evolved less than 2 Mya (Hughes and Eastwood, 2006). Moreover, it is the only legume genus known to be unable to establish mycorrhizal symbiosis. A draft genome sequence of *L. angustifolius* was recently obtained (Hane et al., 2017), showing that all mycorrhiza-symbiotic specific genes have been lost, although this species has retained genes commonly required for mycorrhization and for nodulation. The lupin nodule has unique peculiarities (lupinoid) in which a lateral meristem allows the nodule to grow and surround the root (Figure 1). Beyond *Lupinus* spp., this type of nodule has only been found in some species of *Listia* to our knowledge (Yates et al., 2007; Ardley et al., 2013; Sprent et al., 2017). Using *L. albus* and *Bradyrhizobium* as a model, we described the mode of rhizobia infection of lupin roots and other early steps of nodule development in detail (González-Sama et al., 2004). Bacteria infect the root intercellularly, at the junction between the root hair base and an adjacent epidermal cell, and they invade a sub-epidermal outer cortical cell through structurally altered cell wall regions. This infected cell divides repeatedly and together with uninfected dividing cells, the nodule primordium is formed. Thus, the infected zone of the nodule originates through the division of a single infected cortical cell and therefore, the central zone of the lupin nodules has no uninfected cells.

Despite the advantages associated with the colonization of nitrogen poor environments, the ability of many legumes to nodulate may have evolutionary benefits in terms of alleviating abiotic stress. However, this issue has been little explored. Accordingly, a range of nodulated legumes are found in desert ecosystems and in high altitude areas, suggesting that nitrogen-fixing symbiosis confers an advantage in these ecosystems (Sprent and Gehlot, 2010). Nitrogen-fixing legumes make more efficient used of the available water and their fitness is enhanced in arid and semi-arid climates relative to non-fixing plants (Adams et al., 2016). Some putative adaptations of symbiosis to the environment have been reported and for example, some *Mimosa* species prefer to nodulate with certain rhizobia species rather than others, a preference that may be influenced by soil fertility and pH (Elliot et al., 2009; Garau et al., 2009). The semiaquatic legume *Sesbania rostrata* displays phenotypic plasticity for legume nodulation driven by environmental conditions. Thus, *Sesbania* can develop nodules of the indeterminate or determinate type depending on the environmental conditions (Fernández-López et al., 1998). Similarly, rhizobia infection is via an IT in non-flooding conditions whereas flooding switches the infection mechanism to crack entry, favoring nodulation in conditions of water stress in this legume (Goormachtig et al., 2004). On the other hand, the mode of infection may also be determined by the rhizobia in certain legumes. For example the intercellular via was used by a *S. fredii* strain in *Lotus burttii* (Acosta-Jurado et al., 2016) as well as by a strain of *R. leguminosarum* (Gossmann et al., 2012), whereas a *M. loti* strain enters by IT (Gossmann et al., 2012).

## Organelle-like characteristics of the symbiosome

### Composite origin and differentiation of the symbiosome membrane complex

Several biochemical, genetic, and proteomic studies have set out to characterize the composition of the symbiosome (or peribacteroid) membrane and the peribacteroid space (Whitehead and Day, 1997; Panter et al., 2000; Hinde and Trautman, 2002; Saalbach et al., 2002; Wienkoop and Saalbach, 2003; Catalano et al., 2004; Limpens et al., 2009; Clarke et al., 2014, 2015; Emerich and Krishnan, 2014).

Some membrane microdomain-associated proteins can be found in the SM and they seem to play a key role in the regulation of the nodulation process. Flotillins are markers for membrane microdomains called “lipid rafts.” Flotillin genes are induced during early nodulation events in *M. truncatula* (Haney and Long, 2010). Some of these proteins are involved in infection thread invagination and elongation and they could be involved in endocytosis and trafficking of bacteria and nodule organogenesis (Haney and Long, 2010). Flotillin-like genes are induced in soybean nodules (Winzer et al., 1999) and flotillin-like peptides have been identified and isolated from SM of soybean and pea nodules (Panter et al., 2000; Saalbach et al., 2002). A remorin gene encoding another membrane microdomain-associated protein (MtSYMREM1) is specifically and strongly induced during the rhizobial infection and nodule organogenesis of *M. truncatula* (Lefebvre et al., 2010). This protein was located in plasma membrane of ITs and in the SM and may be a scaffolding protein required for infection and bacterial release into the host cytoplasm (Lefebvre et al., 2010). FWL1 is another interesting membrane microdomain-associated protein identified in soybean symbiosomes (Clarke et al., 2015). FWL1 interacts with remorins, flotillins and other proteins associated with membrane microdomains, regulating legume nodulation (Qiao et al., 2017).

Even at early stages of formation the SM has particular characteristics (Whitehead and Day, 1997), and both the composition and the function of the SM change as it develops (Hinde and Trautman, 2002). In principle, the SM is derived from the plant cell membrane and several plasma membrane markers can be found in the peribacteroid membrane, such as a plasma membrane H^+^-ATPase (Wienkoop and Saalbach, 2003) and the SNARE (N-ethylmaleimide-sensitive factor attachment protein receptor) protein SYP132 (Catalano et al., 2007; Limpens et al., 2009). It is noteworthy that the activation of H^+^-ATPases was also detected in the arbuscular membrane at the AM symbiosis interface (Harrier, 2001).

Symbiosome formation and division induces the activation of the endomembrane system of the host cell (Roth and Stacey, 1989), and it has been proposed that the endoplasmic reticulum (ER) and Golgi vesicles fuse with the SM (Whitehead and Day, 1997; Ivanov et al., 2010; Gavrin et al., 2017). Several proteins from the endomembrane system can be detected in the SM (e.g., cytochrome P450 and a luminal binding protein), and calreticulin, a disulphide-isomerase protein, and some chaperonin-like proteins of the ER have also been identified in symbiosomal fractions and they are probably located in the symbiosome lumen (Saalbach et al., 2002; Wienkoop and Saalbach, 2003; Catalano et al., 2004; Verhaert et al., 2005). Other endomembrane-related proteins in the symbiosome are annexin and syntaxin, which are involved in vesicle transport and secretion, as well as small GTPases involved in the regulation of membrane fusion (Wienkoop and Saalbach, 2003; Catalano et al., 2004; Limpens et al., 2009; Ivanov et al., 2012; Gavrin et al., 2017). It is interesting to note that many of these ER and Golgi proteins, as well as small Rab7 GTPases, have also been found in phagosomes, an organelle compartment of macrophages (Garin et al., 2001; Verhaert et al., 2005), suggesting that symbiosome and phagosome membranes may form in a similar way. Carbohydrate epitopes associated with Golgi-derived glycoproteins and glycolipids have been identified in the inner face of the SM (Perotto et al., 1991). These glycoconjugated molecules, collectively known as the glycocalyx, are involved in physical interactions with the bacterial surface inside the symbiosome and they are important in symbiosome development (Bolaños et al., 2004).

Tonoplast proteins have also been identified in the SM, including a vacuolar H^+^-pyrophosphatase, a vacuolar type H^+^-ATPase (V-ATPase) and an intrinsic tonoplast protein of the Nod26 group (Saalbach et al., 2002; Wienkoop and Saalbach, 2003; Catalano et al., 2004). The presence of active H^+^-ATPases in the SM drives proton accumulation and the establishment of a membrane potential (Whitehead and Day, 1997; Fedorova et al., 1999; Hinde and Trautman, 2002; Clarke et al., 2014). A vacuolar cysteine protease that could be involved in protein turnover and/or the adaptation to changes in cell turgor was also identified in the symbiosome lumen (Vincent and Brewin, 2000; Vincent et al., 2000). This cysteine protease is also involved in nodule organogenesis and function (Sheokand et al., 2005). The vacuolar SNAREs SYP22 and VT111 were also found in senescent symbiosomes (Limpens et al., 2009; Emerich and Krishnan, 2014; Gavrin et al., 2014).

Several proteins originating from mitochondria and chloroplasts are also associated with the SM. Among the chloroplast proteins identified are the peripheral membrane protein F1 ATPase α- and β- subunits, the chloroplast outer envelope protein 34 and a chloroplast nucleoid DNA-binding protein. Mitochondrial membrane proteins have also been found, such as a membrane anion channel (porin) and a nucleotide translocator (malate dehydrogenase), as well as mitochondrial processing peptidases, probably located in the symbiosome lumen (Panter et al., 2000; Saalbach et al., 2002; Wienkoop and Saalbach, 2003; Catalano et al., 2004). Bacterial proteins can also be detected in SMs and the peribacteroid lumen, including several nitrogenase components, chaperones, the α-subunit of bacteroid ATP synthase and others (Whitehead and Day, 1997; Saalbach et al., 2002; Catalano et al., 2004; Emerich and Krishnan, 2014).

The SM is a regulated interface with a key role in nutrient exchange between both symbiotic partners, and different types of proteins and transporters are specifically located at this membrane (White et al., 2007; Clarke et al., 2014; Emerich and Krishnan, 2014). The SM has specific integral membrane proteins, such as nodulin 24 (a glycine-rich protein; Sandal et al., 1992), nodulin 26 (an aquaporin; Dean et al., 1999), and others (Clarke et al., 2015). The sulfate transporter gene (*Sst1*) that is expressed in a nodule-specific manner in *Lotus japonicus*, is essential for nodule symbiosis (Krusell et al., 2005). This transporter seems to reside in the SM (Wienkoop and Saalbach, 2003) and it is thought to transport sulfate from the plant cell cytoplasm to the bacteroids (Krusell et al., 2005). Similarly, a proteomic analysis of the SM from nodules of *L. japonicus* revealed the presence of a putative sucrose transporter of the SUC family (Wienkoop and Saalbach, 2003). More recently, another sucrose transporter (MtSWEET11) was proposed to be located at the symbiosome membrane in *M. truncatula* nodules (Kryvoruchko et al., 2016), suggesting the possible transport of sucrose toward the rhizobia. However, specific transporters for some crucial molecules for nitrogen fixation seem not to be located in the SM. For example molybdenum is a key element for the bacteroidal nitrogenase but the molybdate transporter has not been identified in the SM (Tejada-Jiménez et al., 2017). The sulfate transporter Sst1 (Krusell et al., 2005) could be involved in molybdenum delivery to the symbiosome, as some sulfate transporters can transfer molybdate across membranes (González-Guerrero et al., 2016). Similarly, specific ammonium transporters have not yet been identified in the SM. Although a symbiotic ammonium transporter1 (SAT1) was seen to localize to the SM (Kaiser et al., 1998), it was recently shown that this protein to actually be a membrane-localized basic helix–loop–helix DNA-binding transcription factor involved in ammonium transport (Chiasson et al., 2014). However, ammonium may enter the symbiosome via the aquaporin-like nodulin 26 channel, or through a cation channel that transports K and Na, as well as by diffusion (Tyerman et al., 1995; Hwang et al., 2010; Courty et al., 2015).

The roles and functions of several proteins located at the symbiosome membrane and the peribacteroid space remain unknown (Kereszt et al., 2011; Emerich and Krishnan, 2014). However, the information available provides some markers of the symbiosome membrane identity. Evidence suggests that secretory pathways play an important role in the formation of the symbiosome and perimicrobial compartments, i.e., an exocytosis-related pathway already present in arbuscular mycorrhizal symbiosis. In fact, an exocytotic pathway for endosymbiosis was defined (Ivanov et al., 2012), providing the first evidence that symbiosomes are generated through exocytosis and that they could therefore be considered apoplastic compartments rather than endocytotic compartments. Rhizobia are confined to plasma membrane protrusions, compartments that rapidly increase in surface area and volume due to microsymbiont expansion. Because the plasma membrane is not elastic and it is unable to stretch more than 3%, exocytosis of new membrane material is crucial to increase the membrane's surface area (Grefen et al., 2011). Membrane fusion is achieved through the action of SNARE proteins in the targeted compartment (t-SNAREs) and the vesicle-associated membrane protein (VAMP or v-SNAREs) that form a SNARE complex, small GTPases of the Rab family that control the transport and docking of vesicles to their target membrane, and Ca^2+^-sensors from the synaptotagmin group involved in membrane repair (Catalano et al., 2007; Limpens et al., 2009; Ivanov et al., 2010, 2012; Wang et al., 2010; Gavrin et al., 2016, 2017; Harrison and Ivanov, 2017). Briefly, a plasma membrane t-SNARE (SYP123) is present in the SM throughout the life of the symbiosome (from when the rhizobia is released from the IT to symbiosome senescence) and only when the symbiosome has stopped dividing does the SM acquire a late endosomal/vacuolar marker (Rab7), which persists until senescence. At the onset of senescence, the SM acquires a lytic vacuolar identity due to the appearance of the two vacuolar t-SNAREs (SYP22S, VTI11). These SNAREs allow the symbiosome to fuse and form lytic compartments in which the rhizobia are eventually killed. On the other hand, transporters may have a third, new identity for SM (Emerich and Krishnan, 2014) and it could be speculated that a sulfate transporter like-Sst1 should be considered at this point.

### The symbiosome as a derivative of a lytic compartment

The activity of the vacuolar H^+^-ATPase in the symbiosome membrane leads to the accumulation of protons, which should generate an acidic pH in the symbiosome (Whitehead and Day, 1997; Hinde and Trautman, 2002). Several symbiosome enzymes have an acidic optimum pH, including the proteases, acid trehalase, protein protease inhibitor, and alpha-mannosidase isoenzyme II that are typically found in vacuoles (Mellor, 1989; Panter et al., 2000). In fact, certain mutant and senescent bacteroids are degraded by these proteases and glycosidases, suggesting that the survival of these bacteroids is dependent on them avoiding acid digestion in the symbiosome compartment (Mellor, 1989; Parniske, 2000). As mentioned above, a functional cysteine protease with proteolytic activity has been characterized in the symbiosome lumen (Vincent and Brewin, 2000; Vincent et al., 2000). In 1989, it was proposed that since symbiosomes (which can be considered to be “temporary but independent organelles”) are morphologically different from the plant central vacuole, they may represent organ-specific modifications of lysosomes, analogous to the protein bodies of seeds (Mellor, 1989). Nitrogen activity counteracts the tendency of the ATPase to acidify the lumen of the symbiosome and thus, if the bacteroids stop fixing nitrogen the pH will drop to a level that favors the lysis of the symbiosome. Again, this phenomenon would support the notion of the symbiosome as a modified lysosomal compartment (Brewin, 1991; Hinde and Trautman, 2002).

Symbiosomes do not fuse with lytic vacuoles but they remain as individual units within the cytosol. In fact, it was suggested that vacuolar formation is altered in nodule infected cells in order to allow the expansion of the bacteria in the cytoplasm (Gavrin et al., 2014). Indeed, the vacuoles in infected cells are non-functional and have a neutral pH, or they are degraded (Gavrin et al., 2014, 2016). This facilitates the maintenance of symbiosomes as individual nitrogen-fixing organelles (Limpens et al., 2009; Emerich and Krishnan, 2014).

Rab7 GTPase is thought to be required for the formation of lytic compartments in different organisms (Bucci et al., 2000). In nodules, the plant late endosomal marker Rab7 has been localized in symbiosomes after division stops and it persists until the symbiosome reaches the senescence stage. Therefore, it seems to be involved in symbiosome maintenance (Cheon et al., 1993; Son et al., 2003; Limpens et al., 2009; Clarke et al., 2015). Symbiosome senescence occurs when symbiosomes fuse and form lytic compartments (Hernández-Jiménez et al., 2002; Van de Velde et al., 2006). During senescence, symbiosomes acquire a lytic vacuolar identity, evident through the presence of vacuolar SNAREs and the vacuolar proteins of the HOPS complex at the symbiosome membrane, making it competent for trafficking similar to that of a lytic vacuole (Gavrin et al., 2014).

### The symbiosome behaves like a metabolic organelle

It has been postulated that metabolic innovations may be important for organelle-producing endosymbiosis (O'Malley, 2015). Rhizobia-legume symbiosis depends on the highly regulated exchange of carbon and nitrogen sources, and nutrients, across the bacteroid and SMs. Specific transporters in these membranes that are critical for symbiosis have been identified through transcriptome and proteome analyses (Udvardi et al., 1988; Vincill et al., 2005; White et al., 2007; Clarke et al., 2014). Most rhizobial species only exhibit highly efficient nitrogen fixation when they are endosymbiotic in the host nodule cells. This suggests that the host plant controls rhizobial nitrogen fixation. It was reported that the host plant has overcome the lack of a bacterial gene necessary for symbiotic nitrogen fixation, a homocitrate synthase gene, a key genetic adaptation needed to establish efficient nitrogen-fixing symbiosis in legumes and rhizobia. In *L. japonicus*, a legume host nodule-specific homocitrate-synthase is exclusively expressed in infected cells and it supplies homocitrate to the symbiosome. This tricarboxylic acid is an essential component of the iron-molybdenum co-factor of nitrogenase, although it is not itself required for plant metabolism and it is absent from almost all rhizobia species. This homocitrate makes the nitrogen-fixing activity of the endosymbiont possible and it represents an example of the co-evolution of metabolic pathways in the two symbiotic partners (Hakoyama et al., 2009; Terpolilli et al., 2012). It is interesting to note that photosynthetic bradyrhizobia interacting with *Aeschynomene* legumes can synthesize bacterial homocitrate for free-living and symbiotic nitrogen fixation, and that the plant enzyme is not usually induced. *A. caulinodans*, which form nodules with *S. rostrata*, also has this enzyme. These data suggest that different rhizobia-legume symbioses could have co-evolved differently.

A complex amino acid cycle has been observed in pea nodules, whereby the plant cell supplies amino acids to the symbiosome, which can shut down nitrogen fixation, and in return the latter acts like a plant organelle supplying amino acids back to the plant cell for asparagine synthesis. It has been postulated that this exchange induces mutual dependence, preventing the symbiotic relationship from being dominated by the plant and generating selective pressure for the evolution of mutualism (Lodwig et al., 2003). Further studies into amino acid metabolism suggest that symbiosomes in the indeterminate nodules of pea (carrying *Rhizobium leguminosarum* bv. *viciae* as a microsymbiont) and alfalfa (*E. meliloti*), and in the determinate nodules of soybean (*Bradyrhizobium japonicum*), display metabolic dependence on the host for branched-chain amino acids (Prell et al., 2009, 2010; Dunn, 2014). Thus, symbiosomes become symbiotic auxotrophs and they behave like facultative plant organelles. It was suggested that this enabled the plant to control the degree of bacterial infection (Prell et al., 2009, 2010; Terpolilli et al., 2012; Haag et al., 2013).

Nitrogen fixation is uncoupled from bacterial nitrogen stress metabolism in rhizobia-legume symbiosis, such that bacteria generate “excess” ammonia and release this ammonia to the plant, a case of metabolic integration in this symbiosis (Yurgel and Kahn, 2008). The switching to ammonia synthesis by symbiosomes is accompanied by the switching off of ammonia assimilation into amino acids (Patriarca et al., 2002). Because mature bacteroids deplete nitrogen and release ammonia to the plant without assimilation, it was proposed they could be considered as ammoniaplasts (Oldroyd et al., 2011; Downie, 2014).

### Processing and targeting of symbiosome proteins

The appearance of an organelle-specific protein import mechanism is considered a key step in the conversion of a symbiont into a permanent organelle (Cavalier-Smith and Lee, 1985; Cavalier-Smith, 1992; Theissen and Martin, 2006; Archibald, 2015). Indeed, chloroplasts and mitochondria have developed the specific TIC/TOC and TIM/TOM protein transport systems, respectively. The presence of a signal peptide specific for protein targeting is a distinctive trait of cell organelles. Although strictly referring to targeting in order to reimport proteins back from organelle genes that were transferred to the nucleus, it is interesting to consider the specific targeting of protein products to symbiosomes as an organelle-related process. N-terminal sequence comparisons of some SM proteins, like nodulin 26B and HSP60, suggest that N-terminal signal sequences have been removed from these proteins (Panter et al., 2000). Mitochondrial processing peptidases, homologs of which have been identified in the symbiosome, catalyse the cleavage of leader peptides in precursor proteins, although their function in symbiosomes remains unknown (Catalano et al., 2004). The N-terminal processing of proteins may target them to the symbiosome (Panter et al., 2000; Catalano et al., 2004), although these proteins might be targeted to the ER or Golgi, loosing their signal peptide and later being delivered to the SM via the endomembrane system (Panter et al., 2000). A N-terminal signal peptide in nodulin MtNOD25 specifically translocates this protein to the symbiosomes (Hohnjec et al., 2009), the first clear role for a signal peptide in protein targeting to the symbiosome in nodule infected cells. Other nodulins and calcium-binding proteins from *Medicago, Vicia*, and *Lupinus* carry signal peptides (Hohnjec et al., 2009; Meckfessel et al., 2012), although no conserved N-targeting signal for SM or symbiosome space proteins has yet been identified. Moreover, these symbiosome targeting signal peptides cannot account for the majority of proteins identified in symbiosomes (Hohnjec et al., 2009). Thus, other targeting systems must be available for protein translocation to the symbiosome (Catalano et al., 2004; Clarke et al., 2014).

Vesicle trafficking to the symbiosome via the endomembrane system is not fully understood. It has been postulated that protein delivery to the symbiosome relies on the plant secretory system (Catalano et al., 2007; Limpens et al., 2009; Ivanov et al., 2010; Maunoury et al., 2010; Mergaert and Kondorosi, 2010; Wang et al., 2010) and it is interesting that proteins lacking plastid-targeting signals might also be targeted to the chloroplast via the secretory system (Bhattacharya et al., 2007 and references therein; Mergaert and Kondorosi, 2010). The syntaxin SNARE SYP132, which localizes to the SM (Catalano et al., 2004), may be involved in site-specific vesicle fusion for the delivery of cargo vesicles to the SM (Catalano et al., 2007). Indeed, some tonoplast proteins involved in symbiosome maturation appear to be retargeted to the symbiosome by a mechanism that involves membrane fusion, as observed in infected cells of *Medicago truncatula* nodules (Gavrin et al., 2014, 2017).

### The host legume controls microsymbiont differentiation and proliferation

In *M. truncatula*, the *DMI2* gene that encodes a receptor kinase plays a critical role in the Nod factor signaling cascade during the early stages of nodulation, and it is also a key regulator of symbiosome formation, allowing bacteria to be released from the infection thread into the host cell. In nodules, this kinase is found in the host cell plasma membrane and in the membrane surrounding the ITs. If *DMI2* expression is compromised in plants, infected nodule cells are occupied by large intracellular ITs that do not release the bacteria rather than organelle-like symbiosomes, a phenotype that is reminiscent of the nodules of primitive legumes and *Parasponia* (Limpens et al., 2005; Op den Camp et al., 2011).

In galegoid legumes of the Inverted Repeat Lacking Clade (IRLC), all of which form indeterminate nodules (like *Medicago* and *Pisum*), a legume family of nodule-specific cysteine-rich (NCR) peptides are targeted to the endosymbiotic bacteria. These peptides are responsible for the bacteroid differentiation that involves the induction of endopolyploidy, cell cycle arrest, terminal differentiation, and a loss of bacterial viability. It was recently demonstrated that a nodule specific thyoredoxin (Trx s1) is targeted to the bacteroid, controlling NCR activity and bacteroid terminal differentiation (Ribeiro et al., 2017). The *NCR* gene family is estimated to have appeared between 51 and 25 Mya, the time at which IRLC legumes separated from the other legumes (Lavin et al., 2005; Alunni et al., 2007; Yokota and Hayashi, 2011).

All IRLC species tested induce terminal differentiation of their rhizobia endosymbionts, resulting in different morphotypes. NCR genes were also identified in all these species, although the number of NCR peptides was highly variable, ranging from over 630 in *M. truncatula* to only 7 in the most basal IRLC legume *Glycyrrhiza uralensis* (Montiel et al., 2016, 2017). The nodules of this latter legume lack cationic NCR peptides, which could indicate that the ancestral NCRs were neutral or anionic and that they originated from a single evolutionary event in IRLC legumes (Montiel et al., 2017).

It was proposed that the differentiated polyploid bacteroids might have a more efficient metabolism, like polyploid eukaryotic cells (Van de Velde et al., 2010). NCR peptides are derived from antimicrobial, defensin-related peptides, and these antimicrobial peptides have different mechanisms of action and drive different states of bacteroid differentiation (Haag et al., 2013; Maróti and Kondorosi, 2014; Pan and Wang, 2017). This may be an evolved mechanism by which the host legume dominates microsymbiont proliferation (Mergaert et al., 2006; Mergaert and Kondorosi, 2010; Van de Velde et al., 2010; Maróti and Kondorosi, 2014; Yang et al., 2017). NCR peptides optimize bacteroid metabolism and the nitrogen fixation process (Van de Velde et al., 2010), and they control discrimination against incompatible microsymbionts (Yang et al., 2017). It has also been suggested that this control of bacteroid proliferation by the host plant can avoid the spreading of rhizobia to tissues other than the nodule (Mergaert et al., 2006).

Until recently, it was thought that bacteroids of non-galegoid, non-IRLC legumes, do not undergo terminal differentiation nor is their replication restricted. Indeed, they are comparable to free-living bacteria in cell size, DNA content and proliferation (Mergaert et al., 2006). It is noteworthy that in indeterminate nodules of the mimosoid legume *Leucaena glauca* elicited by *Bradyrhizobium*, no NCR peptides have been detected and the bacteroids display a moderate differentiation phenotype; it is an “intermediate” state relative to that of IRLC and non-IRLC legumes with determinate nodules (Ishihara et al., 2011). The presence of swollen (differentiated) bacteroids has been noted in five out of the six major papilionoid subclades, although each of these subclades also includes species with non-swollen or non-differentiated bacteroids (Oono et al., 2010). Moreover, there was no consistent relationship between nodule type and the host's effects on bacteroid differentiation. Accordingly, it would appear that legumes inducing bacteroid differentiation have evolved independently on five occasions from an ancestral papilionoid legume that hosts non-swollen (non-differentiated) bacteroids (Oono et al., 2010). This repeated evolution of the host's legume traits suggests a possible advantage for the plant in terms of fitness. It has been hypothesized that differentiated bacteroids fix nitrogen more efficiently than non-differentiated bacteroids (Oono et al., 2009, 2010). In fact, Oono and Denison (2010) demonstrated that swollen bacteroids confer net benefits to the host legume due to their more efficient nitrogen fixation and the higher return on the cost of nodule construction (host biomass growth per total nodule mass growth).

It was recently shown that NCR antimicrobial peptides are involved in the permeability of the SM to diverse metabolites. NCR peptides might contribute to metabolic integration between the symbiosome and plant host and in the past, similar antimicrobial peptides may have contributed to the metabolic integration and organellogenesis of mitochondrial and plastid ancestors (Mergaert et al., 2017). This hypothesis emphasizes the importance of metabolic integration in organelle development (see O'Malley, 2015). It was recently discovered that nodules of dalbergioid legume species of the *Aeschynomene* genus (which establish symbiosis with *Bradyrhizobium* spp.) carry polyploid and enlarged bacteroids, and that these plants also express NCR peptides. However, these peptides are not homologous to NCR peptides from IRLC legumes, suggesting an independent evolutionary origin (Czernic et al., 2015).

New plant and bacterial factors that induce bacteroid differentiation remain to be identified (Mergaert et al., 2006; Oono and Denison, 2010; Oono et al., 2010; Van de Velde et al., 2010; Ishihara et al., 2011). A bacterial conserved BacA (bacteroid development factor A) protein that forms an ABC transporter system is produced by rhizobia, and it is required for bacteroid development and survival in IRLC and *Aeschynomene* legumes. BacA may protect rhizobia and bacteroids from the antimicrobial activities of NCR peptides, antagonizing NCR peptides, or it may be involved in the uptake of these antimicrobial peptides by bacteroids (Haag et al., 2013; Guefrachi et al., 2015; Pan and Wang, 2017, and references therein).

It is now assumed that the fate of bacteroids is controlled by the host plant (Mergaert et al., 2006; Maróti and Kondorosi, 2014), although some data suggest that a particular genotype of the microsymbiont might be required, most probably related to their surface polysaccharides. Terminal bacteroid differentiation of *Ensifer fredii* strain HH103 does not take places in nodules of the IRLC legume *Glycyrrhiza uralensis*, (Crespo-Rivas et al., 2016), whereas it does occur when *Mesorhizobium tianshanense* forms the nodules (Montiel et al., 2016). Notably *G. uralensis* is the IRLC legume with the fewest NCR peptides reported to date (Montiel et al., 2017).

Interestingly, species within the genus *Lupinus* may host either swollen (*L. angustifolius*) or non-swollen (*L. albus, L. diffuses*, and *L. bicolor*) bacteroids, suggesting that the effects on bacteroid differentiation might have changed during the evolution of the *Lupinus* genus (Oono et al., 2010). Thus, it is possible that the host legumes have regained non-differentiated bacteroids in these latter three species, because bacteroid differentiation is no longer beneficial (for some unknown reason). Alternatively, some rhizobial strains that nodulate *Lupinus* may have evolved traits to overcome host-induced swelling and the loss of reproductive viability (Oono et al., 2010). To our knowledge, there is no data currently available about NCR peptides or any other similar molecules in the nodules of *Lupinus* (or in other legume nodules with dividing infected cells).

### Division of rhizobia-infected host cells

Infected nodule cells are usually post-mitotic and do not divide further. However, one of the most interesting and quite unusual traits for eukaryotic cells is found in certain legume nodules whose host cells can divide after being infected by rhizobia (Figures 2, 3). The division of infected cells containing symbiosomes has been observed in nodules of *Lupinus* spp. and *Genista tinctoria* (genistoid legumes), and also in certain dalbergioid legumes (e.g., *Arachis hypogea, Stylosanthes* spp., *Sarothamnus scoparius*). All these legumes are infected by *Bradyrhizobium* spp. through epidermal infection or crack infection, and the infected zone of their nodules has no uninfected cells (Chandler, 1978; Chandler et al., 1982; Sprent and Thomas, 1984; Tang et al., 1993; Lotocka et al., 2000; Sajnaga et al., 2001; González-Sama et al., 2004; Kalita et al., 2006; Fedorova et al., 2007). Nodules elicited by *Bradyrhizobium* in the genistoid *Chamaecytisus proliferus* (renamed as *Cytisus proliferus*) also contain dividing infected cells (Vega-Hernández et al., 2001). This is the only elongated indeterminate nodule reported to date without uninfected cells in the central infected zone. Root infection of this legume occurs by a singular intercellular mechanism, and ITs are aborted and do not contribute to infection (Vega-Hernández et al., 2001). Therefore, the division of infected cells appears to be a trait restricted to nodules in which infection is independent of ITs, rather than it being influenced by the type of nodule growth (determinate/indeterminate).

Mitochondria and plastids divide in the plant cytoplasm, and cytoskeletal elements not only secure their distribution and movement but also, their correct partitioning between the daughter cells at cytokinesis (King, 2002; Sheahan et al., 2004). Symbiosomes also have the ability to divide in the host cytoplasm (Figure 1), and the accommodation of endosymbionts in host cells involves microtubule and actin microfilament rearrangements (Whitehead et al., 1998; Davidson and Newcomb, 2001a,b; Fedorova et al., 2007; Timmers, 2008; Gavrin et al., 2015; Kitaeva et al., 2016). The conformation of the cytoskeleton in dividing infected cells of legume nodules has only been studied in *L. albus* (Fedorova et al., 2007). We showed that in the infected cells of *L. albus* nodules, symbiosomes are segregated equally between the two daughter cells when the host plant cell divides, just like other cell organelles, e.g., mitochondria (González-Sama et al., 2004; Fedorova et al., 2007). The cytoskeletal dynamics of infected nodule cells during the cell cycle appear to be relatively normal. In interphase cells, thick cortical arrays of microtubules form a radial network of strands perpendicular to the cell wall to facilitate the migration of organelles and symbiosomes toward the cell periphery (Fedorova et al., 2007). During cell division, symbiosomes concentrate at opposite poles of the cell and do not interfere with the arrangement of microtubules and microfilaments, segregating evenly between the two daughter cells. These cytoskeletal rearrangements in dividing infected cells, along with the detection of an antigen of the molecular motor myosin, suggests that lupin symbiosomes are in contact with and they are driven by the cytoskeleton. Thus, the positioning of symbiosomes in lupin nodule cells seems to depend on the same mechanisms used to segregate genuine plant cell organelles during mitosis (Fedorova et al., 2007). Therefore, in this regard the symbiosome displays significant organelle-like characteristics, unlike symbiosomes from nodules in which infected cells do not divide.

### Considerations about rhizobial genome reduction and gene transfer to the nucleus

It has been established that a key event in the evolution from a free-living bacteria to an organelle is the loss of bacterial genes and their transfer to the nucleus of the plant host, a fate that occurred during mitochondrial and chloroplast evolution (Douglas and Raven, 2003; Archibald, 2015). In rhizobia-legume symbiosis, the presence of duplicated prokaryotic genes in the host genome has yet to be reported, although this possibility cannot be overlooked (Raven, 1993).

In the case of nitrogen-fixing rhizobia, the absence of gene transfer to the nucleus may be due to the low oxygen concentrations required by the nitrogenase enzyme, which would generate poor ROS production and mutation rates (Allen and Raven, 1996). Thus, mutation by ROS generation is unlikely to be an evolutionary driving force in the case of symbiosomes. However, strong ROS production has been detected in nodule host cells and in the symbiosome, the electron transport chain of bacteroids generates superoxide radicals and hydrogen peroxide (Matamoros et al., 2003). Oxidation of nitrogenase and ferredoxin in bacteroids also induces ROS generation (Matamoros et al., 2003). Lipid peroxidation has been detected in the SM during senescence (Puppo et al., 1991) and it could be due to the autoxidation of leghemoglobin (a protein controlling the accurate oxygen level in nodules) that is in direct contact with the SM, as well as to a decline in the activity of antioxidants like superoxide dismutase and catalase that are also present in the bacteroid (Puppo et al., 1991; Matamoros et al., 2003). Moreover, ROS generation induces ultrastructural alterations and senescence of symbiosomes (Puppo et al., 2005; Redondo et al., 2009).

No gene loss or genome reduction has been observed in viable symbiotic rhizobia. Symbiotic rhizobia that do not undergo terminal differentiation are still capable of existing as free-living bacteria. Accordingly, they must be equipped with a number of genes to survive in different environments and to compete with other microorganisms. Moreover, in nodules containing swollen terminally differentiated bacteroids, some non-differentiated bacteria inhabit the apoplastic space and consequently, all the genes necessary for independent life are still retained (Stêpkowski and Legocki, 2001). In fact, rhizobia underwent a genome expansion during evolution (MacLean et al., 2007).

An evolutionary pathway has been proposed in symbiotic systems to shift from free-living organisms to facultative symbiosis and to ecologically obligatory symbiosis, usually involving genome expansion (Provorov et al., 2008). The following step in this evolutionary pathway would be “genetically obligatory symbiosis,” which would involve microsymbiont genome simplification or reduction, and the last stage would be a new organism (Provorov et al., 2008). The availability of more recent molecular data from microbes has driven more in-depth studies into the evolutionary transitions in bacterial symbioses, including rhizobia-legume symbiosis (Sachs et al., 2011a,b). Based on phylogenetic analyses, it was hypothesized that transitions from horizontal to obligate vertical transmission of the microsymbiont are driven by the host, the partner that most benefits from these transitions (Sachs et al., 2011b).

## Concluding remarks

While it has been postulated that organelle development cannot occur in differentiated multicellular organisms (McKay and Navarro-González, 2002), the information presented in this review suggest that the symbiosome might well be considered a step in the co-evolution of legumes and rhizobia toward a nitrogen-fixing organelle. Symbiosomes display features that favor their consideration as nitrogen-fixing organelles, including the host cell's control of microsymbiont proliferation and differentiation, the composite origin and differentiation of the symbiosome membrane, the retargeting of the host cell's proteins, or their metabolic behavior. In some legume nodules, such as lupin nodules, host cells seem to perceive their symbiosomes as entities equivalent to their own real organelles. As such, division of infected cells involves the normal cytoskeletal arrangements of regular dividing plant cells, allowing symbiosome segregation into the daughter cells in the same manner as other cell organelles. Symbiosomes in nodules with dividing infected cells might represent a crucial step in the evolution toward real organelles. Nodules with dividing cells form in evolutionarily older legumes in which rhizobial infection does not occur via ITs. In this context, distinct evolutionary routes cannot be ruled out for nodules with non-dividing infected cells and their symbiosomes. In fact, the differences among nodules range from those with nitrogen-fixing ITs and no symbiosomes, to those with infected cells that are able to divide with an organelle-like segregation of the symbiosomes. These could be considered different events or steps in the evolution toward the nitrogen-fixing organelle. In any case, they represent different outcomes or stages in the co-evolution processes, which might or might not continue.

## Author contributions

TC, EF, JP, and ML wrote the manuscript. All the authors read and approved the final version of the manuscript.

### Conflict of interest statement

The authors declare that the research was conducted in the absence of any commercial or financial relationships that could be construed as a potential conflict of interest. The reviewer OS and handling Editor declared their shared affiliation.
